# Identifying and exploring the self-management strategies used by childhood cancer survivors

**DOI:** 10.1007/s11764-020-00935-2

**Published:** 2020-11-06

**Authors:** Morven C. Brown, Anna Haste, Vera Araújo-Soares, Roderick Skinner, Linda Sharp

**Affiliations:** 1 Population Health Sciences Institute, Newcastle University, Sir James Spence Institute, Royal Victoria Infirmary, NE1 4LP, Newcastle upon Tyne, UK; 2 Newcastle University Centre for Cancer, Newcastle University, Newcastle upon Tyne, UK; 3 School of Social Sciences, Humanities and Law, Teesside University, Middlesbrough, UK; 4 Translational and Clinical Research Institute, Newcastle University, Newcastle upon Tyne, UK; 5 Newcastle upon Tyne Hospitals NHS Foundation Trust, Newcastle upon Tyne, UK

**Keywords:** Childhood cancer, Survivor, Self-management, Qualitative

## Abstract

**Purpose:**

Childhood cancer survivors (CCSs) are at increased risk of chronic health problems. Effective self-management could help CCSs cope with the challenges that accompany survivorship and reduce their risk of developing further health problems. There is little evidence about the extent to which CCSs engage with self-management and the specific strategies they use. This study aimed to identify and explore the strategies that CCSs use to manage the consequences of cancer.

**Methods:**

Twenty-four CCSs were recruited via follow-up clinics. Participants completed a semi-structured interview which was audio-recorded and transcribed. Directed content analysis was used to identify self-reported self-management strategies and categorise them into main self-management types.

**Results:**

CCSs reported 118 specific self-management strategies which fell under 20 main self-management strategy types. All CCSs reported using several main self-management strategy types and specific self-management strategies. Main strategy types used by all CCSs were “adopting a healthy lifestyle”, “self-motivating”, “using support”, “reasoned decision-making” and “creating a healthy environment”. The most common specific self-management strategies were “receiving family support” (*n* = 20) and “attending follow-up and screening appointments” (*n* = 20).

**Conclusions:**

This is the first study which has enabled CCSs to self-report the numerous strategies they employ to look after their health and well-being, contributing to a more comprehensive picture of self-management in CCSs.

**Implications for Cancer Survivors:**

These findings may increase healthcare professionals’ awareness of the many ways in which CCSs manage their health and is a valuable first step in the development of a supported self-management intervention for CCSs in follow-up care.

**Electronic supplementary material:**

The online version of this article (10.1007/s11764-020-00935-2) contains supplementary material, which is available to authorized users.

## Introduction

As a consequence of advances in paediatric cancer treatments, there are now approximately 40,000 childhood cancer survivors (CCSs) in the UK [1], and up to 500,000 CCSs in Europe [2]. CCSs are at risk of a range of late-adverse effects of treatment; in excess of 60% of CCSs will develop at least one chronic health problem [3, 4], whilst approximately 40% will experience neurocognitive deficits [5]. Survivors may also be at risk of experiencing poor psychological health and of encountering challenges in relationships, education, and employment [6]. Therefore, regular ongoing multidisciplinary follow-up care is generally considered essential to monitor and manage CCSs’ biopsychosocial health [7]. However, post-treatment, the frequency of follow-up appointments generally decreases, and at approximately 5 years after treatment, CCSs often enter long-term follow-up (LTFU) care. CCSs at lowest risk of late-adverse effects may have no routine specialist follow-up [8]. Moreover, long-term CCSs may also be discharged from care. Therefore, increasing demands are placed on the survivor and their family to manage their health and well-being. It is possible that effective engagement by CCSs in self-management could help them to reduce their risk of developing adverse effects of cancer or its treatment and improve how they cope with the challenges associated with surviving cancer.

Self-management is a well-recognised concept in chronic diseases. It involves the individual monitoring the condition and using a range of strategies—such as decision-making, problem solving, or resource management—to maintain a sense of wellness, rather than illness [9]. Effective self-management of chronic illness can result in positive effects on disease and symptom control, disease knowledge, self-efficacy, quality of life, and self-management behaviours such as medicine adherence and physical activity [10, 11].

In cancer, self-management has been defined as an “awareness and active participation by the person in their recovery, recuperation, and rehabilitation to minimise the consequences of treatment, promote survival, health and well-being” [12]. A growing evidence base indicates that many survivors of cancers diagnosed in adulthood are open to, or engage with, a wide variety of self-management strategies [13, 14]. In addition, there is emerging evidence that self-management interventions have the potential to improve outcomes in cancer survivors [15]. However, results are difficult to interpret due to heterogeneity and methodological limitations with current interventions [16]. In addition, the provision of self-management support in routine care for survivors lags behind that of chronic diseases [15]. Despite this, it is recognised that cancer survivors should be supported to self-manage which, in the case of young cancer survivors, should lead to enhanced autonomy and empowerment [12].

To inform the need for, and development of, supported self-management approaches for CCSs, there is a need to better understand self-management among CCSs. It is possible that—because of their age and life stage—CCSs’ willingness and ability to engage with self-management, and the strategies they adopt, may differ from those of adults diagnosed with cancer in middle or older age. Evidence suggests that CCSs may have poor knowledge about their disease, its treatment, and any potential health implications [17], and often lack concern about their future health [18]. Moreover, CCSs have to self-manage their health against the background of the challenges, expectations, and important developmental changes typically associated with adolescence and young adulthood [19].

Evidence is limited on self-management in CCSs. A few studies have identified self-management strategies used by adolescents and young adults in active treatment [20], and the self-management needs of survivors of cancers diagnosed in adolescence and young adulthood [21, 22]. Only one study appears to have examined self-management strategies used by young adult survivors of childhood cancer post-treatment and that study invited survivors to endorse which of a list of 16 pre-defined strategies they used to manage specific late-effects [23]. Moreover, whilst several frameworks have been developed to identify and categorise the self-management strategies used by individuals with chronic illnesses, including cancer [13, 14, 24], the extent to which these capture self-management strategies used by CCSs is unknown.

This study aimed to identify strategies that young adult CCSs use to manage the consequences of cancer in their everyday lives and explore how CCSs describe using these strategies and the psychological processes underlying these reported strategies.

## Methods

### Design

The study used qualitative methodology (semi-structured interviews) to collect deep and detailed data of CCSs’ experiences of self-management.

### Participants and recruitment

Participants were young adult CCSs who attended outpatient follow-up clinics at a principal treatment centre for childhood cancer in the North East of England. Individuals were eligible if they had been diagnosed with haematological cancer, or central nervous system (CNS) or other solid tumour at age 18 years or under, were currently at least 18 years old, at least 3 years from diagnosis, no longer on treatment, and free from cancer for at least 1 year; could communicate in English to a level which would allow them to participate in an interview; and would be able to provide informed consent.

To identify potentially eligible survivors, consultants and nurse specialists screened patient lists of forthcoming clinics. Purposive sampling was used to ensure participants had a range of diagnoses (haematological malignancy/CNS tumour/other solid tumour) and times since diagnosis (< 5 years/≥ 5–10/> 10 years). Survivors considered eligible were provided with brief information about the study by mail in advance of their clinic appointment, or verbally from their consultant/nurse specialist whilst at the clinic.

Whilst at clinic, survivors who were potentially interested in the study were asked if they consented to their contact details being passed onto the researchers (MB and AH) or, if a researcher was available, asked if they would agree to briefly meet them. Survivors were provided with an information sheet, were able to ask questions, and were able to state if they wished to be contacted further regarding the study or not. After at least 2 days, the same researcher subsequently contacted the interested survivor by phone to ask if they would be willing to take part in the study and arrange a convenient date and time for the interview. The study was approved by the London City & East Research Ethics Committee (REC reference 16/LO/2267).

### Data collection

Interviews were conducted by MB and AH, both of whom are health psychologists trained and experienced in qualitative research. Interviews were conducted either face-to-face (in a private room within a university research department or at the survivor’s home) or by telephone, as the interviewee preferred. All participants provided either written (for face-to-face interviews) or verbal consent (for telephone interviews; this was audio-recorded).

Interviews were semi-structured using a topic guide, which comprised open questions informed by literature review, theory, and expert knowledge [25, 26]. The topic guide was piloted prior to data collection and modified as required. The guide was then used flexibly throughout the interview process so that any new issues raised by participants could be added to the guide and explored further in subsequent interviews; a copy of the guide is available from the authors on request. Participants were first invited to tell the interviewer a little about themselves, their disease, and treatment history; subsequently, the interviewer explored participants’ views on their own health, issues they experienced with their health, and how they looked after their health and any problems they encountered in doing so. Finally, participants were invited to raise any additional issues which they felt were important to the issue of looking after their health. Participants were offered a £20 shopping voucher to thank them for their time, reimbursement of any travel expenses, and a Children’s Cancer and Leukaemia Group (CCLG) “healthy lifestyle factsheet” [27].

Recruitment continued until data saturation as per the principles defined by Francis et al [28]. Interviews lasted between 35 and 175 min (mean = 78 min) and were audio-recorded.

### Data analysis

Interviews were transcribed verbatim. Transcripts were anonymised and pseudonyms applied. The current analysis focused primarily on identifying and exploring self-management strategies reported by CCSs, i.e. strategies they actively engaged in to improve or maintain their health and well-being. Therefore, directed content analysis which uses previous research findings to inform the structure of the analysis was employed [29–31]. By developing a categorisation matrix, the data is analysed deductively and coded to predetermined categories [30, 31]. However, directed content analysis also enables the identification of newly established categories through the principles of inductive content analysis, thus allowing previous findings to be both supported, refined, and extended in a new context [31].

Dunne et al.’s (2017) framework of self-management strategy types [13], derived from interviews with head and neck cancer survivors who had completed treatment, informed the categorisation matrix; this framework builds upon, and extends, previous frameworks of self-management strategies in cancer survivors [14] and patients with chronic illness [24]. The initial categorisation matrix consisted of categories (20 main self-management strategy types), which were then described through sub-categories (77 specific self-management strategies). Concurrent inductive analysis was undertaken to ensure identification of any additional self-management strategies used by CCSs but not included in Dunne et al.’s framework [13].

Two researchers (MB and AH) independently read and re-read a sample of transcripts (*n* = 6 out of 24) and coded relevant text to the framework categories. Any text which appeared to be describing a self-management strategy but which could not be coded into existing categories was coded into a new category and given a suitable descriptive coding label. The researchers met to discuss similarities, differences, difficulties, and any newly identified categories. Coding rules were developed to help distinguish between categories, with anchor examples providing concrete examples of each specific strategy [32]. Where necessary, the definitions for main strategy types and specific strategies in the framework were amended slightly to more clearly reflect the context of CCSs. The remaining transcripts were analysed by MB with a rechecking and reworking of types and strategies throughout the process to increase reliability [32]. As analysis progressed, findings and uncertainties were discussed among the team. Analysis was facilitated by NVivo version 11. To describe the extent to which CCSs use self-management, we report the frequencies with which main strategy types and specific strategies were used. Illustrative quotes are also provided. A description of the most common main strategy types is also presented.

## Results

### Participants’ characteristics

Twenty-four of the 51 invited eligible CCSs were interviewed. The characteristics of the participants are shown in Table 1. The median age was 23.9 years and 14 (58%) were female. Sixty-two percent (*n* = 15) had haematological cancer, 17% (*n* = 4) had CNS tumours, and 21% (*n* = 5) had other solid tumours. The mean age at diagnosis was 11.0 years, and mean time since diagnosis was 11.6 years. All were in follow-up care.

**Table 1:** Participant characteristics

Characteristic		
Gender, *n* (%)	Female	14 (58%)
	Male	10 (42%)
Diagnosis, *n* (%)	**Haematological**	**15 (62%)**
	*Leukaemia*	*8*
	Acute lymphoblastic leukaemia	3
	Acute myeloid leukaemia	2
	Acute promoyelocytic leukaemia	2
	Myelodysplasia	1
	*Lymphoma*	*7*
	Hodgkin’s lymphoma	4
	Non-Hodgkin’s lymphoma	3
	**Central nervous system and brain tumours**	**4 (17%)**
	Ependymoma	2
	Craniopharyngioma	1
	Low-grade glioma	1
	**Other solid tumours**	**5 (21%)**
	Ewing’s sarcoma	1
	Osteosarcoma	1
	Rhabdomyosarcoma	2
	Breast	1
Treatment received, *n* (%)	Chemotherapy	20 (83%)
	Radiotherapy	11 (46%)
	Surgery	8 (33%)
	Bone marrow transplant	5 (21%)
	No treatment	1 (4%)
Age at study (years) Mean (range)	23.9 (18–33)	
Age at diagnosis (years) Mean (range)	11.0 (2–18)	
Time since diagnosis (years) Mean (range)	11.6 (3–27)	

### Use of self-management

All 20 main self-management strategy types were evident in the data, and no new strategy types were identified. A total of 118 specific self-management strategies were reported (Table 2). All CCSs reported the use of several main self-management strategy types (median 13; range 6–18), and within these, multiple specific self-management strategies (median 47; range 20–70), to aid rehabilitation from cancer, manage any current conditions and care for their health and well-being. The final definition of each strategy type and further anchor examples for each specific self-management strategy reported by participants is shown in Supplementary File 1.

**Table 2:** Self-management strategy types and specific self-management strategies

Self-management strategy type	Specific self-management strategy
Acceptance	Accepting cancer and its consequences
	Accepting new health behaviours
	Accepting social difficulties
Activity-based coping	Pursuing an existing hobby/activity
	Taking up a new hobby/activity
Adopting a healthy lifestyle	Adopting a healthy diet
	Avoiding negative health behaviours*
	Being physically active in everyday life*
	Ensuring personal hygiene*
	Exercising
	Drinking more water*
	Meditating
	Reducing negative health behaviours
	Taking medication*
	Taking vitamins and minerals*
	Sleeping well*
Behavioural avoidance	Avoiding activities that may cause harm
	Avoiding situations that may cause harm*
	Avoiding contact with others for possible infection
	Avoiding uncomfortable social encounters
Cognitive avoidance	Avoiding finding out too much
	Avoiding thoughts about cancer and its consequences
	Dealing with (in)fertility at the right time*
	Distracting oneself by keeping busy
Conserving emotional energy	Caring less about what others think^§^
	Having time to yourself*
	Letting emotions out*
	Minimising stress
	Switching off*
	Using sleep*
Conserving physical energy	Reducing activities
	Reducing workload^§^
	Taking a break
Creating a healthy environment	Acquiring knowledge about cancer, treatment and late effects and available support (*adapted from acquiring knowledge about condition and available support*)
	Attending follow-up and screening appointments*
	Collecting materials to aid self-management
	Ensuring reliability of health information on the internet*
	Learning self-management skills
	Obtaining resources to aid self-management*
	Relationship-building with health practitioner^§^
	Utilising skills for independent living*
	Valuing and respecting relationship with cancer care team *
Goal and action setting	Coping planning*
	Planning daily activities
	Priority-based planning^§^
	Setting future goals
	Setting up facilitating conditions
Managing others	Avoidance of negative relationships*
	Being assertive in social encounters
	Being open with others and cancer and its consequences
	Keeping others happy
	Protecting others from harm
Meaning-making	Appreciating health more
	Appreciating life more
	Appreciating support
	Appreciating the importance of family
	Appreciating the severity of one’s cancer history*
	Becoming more altruistic
	Changing one’s image^§^
	Finding meaning in work
	Giving back*
	Taking every day as it comes*
	Wanting to give something back*
Positive appraisal	Benefit finding
	Downward comparison
	Reinterpreting negative consequences
Proactive problem solving	Acting to prevent further complications
	Adaptive approaches to ongoing physical consequences of cancer and its treatment
Reasoned decision-making	Considering benefits of positive health behaviours*
	Considering pros and cons of self-management
	Evaluating effectiveness of self-management
	Thinking objectively about negative health behaviours
	Thinking objectively about negative thoughts and emotions
Seeking normality	Balancing life with health needs*
	Carrying out tasks to the best of one’s ability*
	Choosing when and to whom to disclose cancer history*
	Focusing on doing normal activities^§^
	Focusing on getting back to work^§^
	Gaining independence*
	Maintaining independence^§^
	Regaining strength*
	Returning to normal*
	Testing oneself^§^
	Trying to fit in*
Self-monitoring	Knowing your body*
	Monitoring emotions
	Adapted from monitoring symptoms and side-effects
	Monitoring general health
	Monitoring health behaviours*
	Recognising one’s own limits*
	Monitoring relationship with health professionals^§^
Self-motivating	Being healthy for sake of one’s family*
	Challenging yourself*
	Developing confidence and self-efficacy*
	Drawing on spiritual resources^§^
	Drawing strength from past experiences*
	Employing a determined attitude
	Encouraging oneself
	Focusing on milestones of survivorship
	Interacting with others*
	Maintaining a positive outlook
	Not dwelling on the past*
	Persevering with healthy behaviours
	Recognising the need for motivation and discipline*
	Rewarding oneself^§^
	Taking responsibility for own health*
	Treating illness as a project^§^
	Wanting to look good*
	Wanting to stay in good health*
Self-sustaining	Following health practitioner’s advice
	Incorporating self-management behaviours into daily routine
	Maintaining medical equipment^§^
	Customising dietary practices^§^
	Keeping busy to avoid negative behaviours*
Using sense of humour	Finding humour in others’ reactions
	Laughing about cancer and its consequences
	Using humour to hide insecurities*
Using support	Companionship from pet
	Drawing support from similar other
	Having someone to talk to*
	Receiving formal support
	Receiving support from charities and organisations*
	Receiving support from educational provider*
	Receiving support from family^†^ *(adapted from receiving support from family and friends)*
	Receiving support from friends^†^ *(adapted from receiving support from family and friends)*
	Receiving support from cancer care team*
	Receiving support from partner^†^ *(adapted from receiving support from family and friends)*
	Receiving support in the workplace*
	Seeking formal help
	Seeking support from family^†^ *(adapted from seeking support from family and friends)*
	Seeking support from friends^†^ *(adapted from seeking support from family and friends)*
	Seeking support from cancer care team*
	Seeking support from partner^†^ *(adapted from seeking support from family and friends)*

*New specific strategy identified in the CCSs data

^†^Original specific strategy has been sub-divided into new categories

^§^Original specific strategy not identified in CCSs data

### Revisions to previous framework

Of the 77 specific self-management strategies in the analysis framework, 62 were reported by CCSs. The labels for two of these 62 previously reported strategies were modified slightly to ensure greater relevance to the context of CCSs (*acquiring knowledge about condition and available support* became *acquiring knowledge about cancer*, *treatment*, *late-effects*, *and available support*; *monitoring symptoms and side*-*effects* became *monitoring for symptoms of cancer and late-effects*). A further two of these original strategies were evident in the data (*receiving support from family and friends* and *seeking support from family and friends*), but the depth of data reported by CCSs enabled these two specific strategy types to be revised into six separate categories which encompassed support from family, friends, and partners. Example quotes are shown in Supplementary File 1.

No supporting evidence was found for 15 of the original strategies. An additional 52 novel specific strategies (within 15 of the main strategy types) were identified (Table 2 and Supplementary File 1).

### New specific self-management strategies reported by CCSs

Within the main strategy type “adopting a healthy lifestyle”, CCSs reported engaging in seven additional health behaviours (e.g. *taking medication*, *drinking more water*). As well as the original specific strategy of *exercise*, a new strategy of *being physically active in everyday life* was also identified. Similarly, whilst CCSs reported the original specific strategy of *reducing negative health behaviours*, they also described complete *avoidance of negative health behaviours*.

For the strategy type “conserving emotional energy”, four new specific strategies were reported by CCSs (*having time to yourself*, *letting emotions out*, *switching off*, *using sleep*). For the strategy type of “creating a healthy environment”, CCS reported five additional specific strategies of *attending follow*-*up and screening appointments*, *ensuring reliability of health information*, *obtaining resources to aid self-management*, *utilising skills for independent living*, *and valuing and respecting relationship with cancer care team*.

Three new specific strategies were coded in “meaning-making”: *appreciating the severity of one*’*s cancer history*, *giving back*, and *taking every day as it comes*. Seven new specific strategies were utilised by CCSs to try to live as normal lives as possible (e.g. *balancing life with health needs*, *trying to fit in*, and *gaining independence*). Three additional strategies by which CCSs undertook active “self-monitoring” of their health (*knowing your body*, *monitoring health behaviours*, and *recognising one*’*s own limits*) were identified. Furthermore, an additional ten “self-motivating” strategies were reported (e.g. *being healthy for the sake of your family*, *drawing strength from past experiences*).

Within the main strategy type of “using support”, six new specific strategies were established for other important sources of support reported by CCSs (e.g. healthcare professionals, charities, educational providers), as well as a more generic category for CCSs who recognised the importance of *having someone to talk to.*

An additional specific self-management strategy was reported in each of the following main strategy types: behavioural avoidance; cognitive avoidance; goal and action setting; managing others; reasoned decision-making; self-sustaining; and using humour. Illustrative quotes for these new specific strategies can be found in Supplementary File 1.

### Most frequently reported main and specific strategies

The five most frequently reported main strategy types, reported by all CCSs, were “adopting a healthy lifestyle” (*n* = 24), “self-motivating” (*n* = 24), “using support” (*n* = 24), “reasoned decision-making” (*n* = 24), and “creating a healthy environment” (*n* = 24); these are described further below. The specific self-management strategies most commonly identified in the data were *receiving support from family* (*n* = 20), *attending follow-up and screening appointments* (*n* = 20), *thinking objectively about negative health behaviours* (*n* = 19), *exercising* (*n* = 18), and *considering the benefits of positive health behaviours* (*n* = 18).

### Adopting a healthy lifestyle

Most survivors engaged in a range of sports and activities (e.g. football, wheelchair basketball, swimming, gym) to keep fit, and walked for exercise (e.g. walking the dog). Several CCSs also commented that they were constantly on the move in their everyday lives, mostly as a result of their jobs.

“I’m running up and down 4 flights of stairs at least 7 times a day, you know it’s not that I’m not unfit you know. If anything working in that [shop] has made me fitter.” Chris, 25 years old

CCSs also described attempts to improve how they eat (e.g. not missing breakfast, eating three meals a day, controlling portion size, reducing snacking between meals) and what they eat (e.g. eating a balanced diet).

“I used to just eat until I couldn’t physically eat any more, now I will just eat until I feel slightly full and stop right there and it really helps a lot.” Abigail, 18 years old

To protect their current and future health, most CCSs reported reducing or limiting negative health behaviours, mainly limiting alcohol consumption but also limiting consumption of sugary drinks and sun exposure. Avoidance of negative health behaviours (cigarette smoking and illegal drugs) was also common.

“I don’t drink alcohol, I don’t smoke, I don’t do drugs, so they’re the big three.” Daisy, 23 years old

Most survivors also reported taking a range of prescribed medications to maintain their physical and mental well-being (e.g. treatments for anxiety, depression, diabetes insipidus, diabetes mellitus, epilepsy, growth hormone deficiency, hypothyroidism, irritable bowel syndrome, pain, hormone replacement therapy as well as aspirin and penicillin).

“I’m taking the stomach tablets, as I said, I’m doing that, but I think I used to take penicillin or something similar on a daily basis, but I don’t want to take tablets all the time. The stomach ones, I can feel the real benefits, so I’ll do it.” Matthew, 33 years old

A small number of survivors reported other strategies such as ensuring they were regularly washing their hands, drinking enough water, taking vitamin and mineral supplements, meditating, and ensuring enough sleep.

### Self-motivating

Survivors felt it was important for them to take responsibility for their own health. For several, control over their health was considered important in order to prevent future illness and there was a recognition that as young adults, they themselves were responsible for this and not their healthcare professionals or parents.

“I am 21 now so you have got to sort it out yourself in your head and say, ‘I can do this’.” Oonagh, 21 years old

Participants acknowledged that motivation is key for undertaking positive health behaviours and achieving health goals. They described how wanting to maintain their health and look good motivated them to look after their health. Other motivational strategies included self-encouragement and determination. Some survivors commented that despite struggling with some health behaviours initially (e.g. taking medication, doing exercise), perseverance meant that these behaviours were now part of their life.

“I don’t necessarily think about my medication because it’s like err, it just … sometimes I do it without even realising that I’ve done it erm, it’s like a bit of like an instinct now, like I don’t even look at the buttons anymore, I just know what to do without looking.” Abigail, 18 years old

Strategies which helped CCSs to stay motivated included a realisation of their strength and resilience, maintaining a positive outlook, not dwelling on the past, challenging themselves, and developing confidence. For some, their self-motivation improved as they moved further beyond treatment; they considered that this meant there was less chance of the cancer returning. Social interaction was viewed as being important to keep motivated and engaged.

“I’ve more and more started saying hello to people. And that little bump in the morning of talking to random strangers who are walking dogs, is actually quite nice. So that’s a like little bit of holistic therapy I’ve been doing for meself.” Chris, 25 years old

### Using social support

Parents and, for some survivors, their partners provided emotional and practical support (e.g. preparing meals). For some, parents also provided financial support. CCSs described how parents, partners, friends, and healthcare professionals within their cancer care team encouraged them to take care of their health. CCSs described how support provided by their cancer care team included advice and information about follow-up care, services available, and emotional support.

“I feel better seeing them [cancer follow-up team] because I do get like I said paranoid at times with my health and that, so when I do go to them they check me so it gives me that ease that ok they’ve checked me there’s nothing there.” Laila, 18 years old

Whether it was a parent, friend, or a professional, many survivors acknowledged it was just good to have someone who you can talk to, should you need it. Friends gave the survivors someone to laugh and talk with and were often important sources of motivation and support when undertaking planned exercise. Several survivors received support from those with similar experiences to whom they could relate, including other cancer survivors and people with other conditions or disabilities, or other young people who also felt “different”:

“I think I’ll only ever need him [a friend] because he’s different as well. He’s not disabled, but he’s different in his own way … when we went out we were like, “we’ll face it together”, do you know what I mean? He was, like ‘I don’t care about the way you walk’. I always said to him, ‘I don’t care about the way you look'. So, we did it together.” Rosie, 22 years old

Participants also sought or received formal support from their general practitioner and from psychologists, psychiatrists, counselling services, and physiotherapy and from charitable organisations such as Teenage Cancer Trust. A few also reported encouragement and practical support from teachers and their school or university or place of work. A few mentioned companionship from a pet, and for these survivors, their dog was viewed as a key source of emotional support.

“You can’t really feel rubbish when you’ve got a dog around, at least I don’t think so anyway.” Matthew, 33 years old

### Reasoned decision-making

Most CCSs described their views about a range of health behaviours and how these influenced their reasons to engage in, or abstain, from them. The positive physical and psychological benefits of physical activity/exercise were widely reported: survivors felt more confident, happier, and refreshed afterwards. Positive health behaviours (such as exercise and a healthy diet) were seen to have immediate effects on well-being as well as being potentially beneficial for long-term health:

“I’d definitely say swimming is the best one because it uses every muscle and it’s keeping yourself healthy and stuff. It just calms you cos you feel a lot better.” Joanne, 21 years old

Conversely, the harmful effects of negative health behaviours were offered as reasons to not engage in these behaviours by most, but also as reasons to stop by those who felt they consumed too much alcohol or who were current smokers:

“But I need to stop smoking because … you … just can’t run for toffee if you smoke so.” Alice, 32 years old

In addition to acknowledging the benefits of positive health behaviours, several CCSs also described weighing up of the pros and cons associated with self-management. The disadvantages of taking action to self-manage their health included the “hassle” of taking medications, the potential implications of medications on future pregnancies, the financial cost of looking after yourself, and anxieties associated with attending follow-up and experiencing “scanxiety” whilst waiting for scan results. For a few survivors, potential cons of physical activity were that exerting yourself could lead to feelings of fatigue, and for two survivors, there was uncertainty about the risks involved in taking part in particular activities and concerns about safety:

“So it’s too big a risk really, just anything like that [playing football] and I’m not much of a risk-taker. I’d rather just play it safe and avoid the activity, rather than risk getting hurt and something bad happening.” Beth, 25 years old

Some CCSs who experienced negative thoughts and emotions, reported trying to think objectively about these. They described ways in which they recognised they had negative patterns of thoughts which, if focused on, could lead to a worsening of mental health. Survivors described efforts to re-direct their thoughts in a more positive way, rationalising their thoughts and being kinder to themselves:

“Try to rationalise things more. Try and relax more. It’s easier said than done. Try and be more positive and not scrutinise things and go over things so much. I massively self-doubt and am very pessimistic.” Quinn, 27 years old

### Creating a healthy environment

Most survivors described attending their follow-up appointments as a way of self-managing their health. Many reported that they had received written information from their clinical team, such as a treatment summary and care plan or information on healthy lifestyles:

“I got a little leaflet thing, it’s like a few A4 pages, it erm, it’s basically a summary of my all my medication and that and history and all that, it’s just literally just bullet points telling me what not to do.” Tara, 18 years old

Some talked about actively asking their healthcare provider about their diagnosis, treatment, and its implications, and others spoke about seeking information on the Internet, particularly as they aged (with a few commenting on the importance of only using health information from reliable online sources). Others discussed the relationship they had with their oncologist/haematologist and the wider healthcare team: because they had often known these team members throughout their illness trajectory, this relationship was valued and trusted.

“I mean I wouldn’t be here today if it wasn’t for their hard work and you know what I mean for them so I wouldn’t … he didn’t let me down so can I really afford to let them down? No.” Hugh, 26 years old

Survivors also reported that they themselves had secured resources such as gym membership, gym equipment, and cookbooks to aid self-management. Several survivors reported how they utilised skills for independent living such as cooking or understanding nutrition labels on pre-packed foods and two described learning particular skills (how to take their medication and techniques for stress management).

## Discussion

CCSs are at risk of a wide range of medical, neurocognitive, psychological, and social problems which, for some, persist or worsen over time or may even become more complex because of the development of new late-effects or health conditions as they age. Encouraging and facilitating self-management is (or should be) a feature of high-quality LTFU care for CCSs [7]. In order to provide this, and to inform the need for, and development of, interventions to promote and support self-management, it is essential to first understand the extent to which CCS engage in self-management and what strategies they use.

This study identified the self-management strategies and processes that CCSs employ in order to actively care for their health and well-being. Approaches reported by all CCSs included the use of social networks for support, the adoption of healthy behaviours, strategies to increase motivation to engage in effective self-management, the use of objective decision-making processes to form views on health behaviours, and attempts to create environments favourable for self-management. Survivors acknowledged that they adopted these strategies not only to aid rehabilitation from cancer treatment and to manage any current conditions but to also maintain and protect their current and future health.

Use of qualitative methods enabled CCSs to self-report and describe the numerous and varied strategies they employ to look after all aspects of their well-being. Classification of reported strategies was informed by previous self-management typologies and frameworks for cancer survivors and chronic illness [13, 14, 24]. By using this approach, we identified 20 main strategy types and 118 specific strategies used by CCSs. These strategies spanned across psychological, social, and behavioural approaches and many have not been described in CCSs previously.

Although all the main strategy types proposed by Dunne (2017) in the context of adults diagnosed with cancer were also identified in the responses of CCSs [13], the prevalence of the strategies differed (to the extent to which prevalence can be compared within qualitative studies). Considerations of the benefits and harms of health behaviours, as well as attempts to adopt positive health behaviours, were more commonly reported in CCSs than in adult cancer survivors. Youth is a critical period for developing attitudes and exploring health behaviours which, if established, can then continue into adult life [33]. However, health behaviours are influenced by a wide range of factors and adolescence is also known as a time for risk-taking [19]; therefore, despite the shared view that particularly negative health behaviours such as smoking can cause harm, it is well-known that this does not always translate into abstinence [34]. Similarly, despite the fact that CCSs commonly reported use of strategies to increase motivation to self-manage, it is worth noting that motivation does not always lead to adoption or maintenance of health behaviours; this is the so-called intention-behaviour gap [35]. Therefore, although we have identified the strategies that CCSs report using, we do not know how often they were engaged in, or whether they were effective.

Popular strategies to increase self-motivation reported by participants included maintaining a positive outlook, encouraging oneself, drawing strength from past experiences, and employing a determined attitude. These psychological strategies are common in chronic illness and among adult cancer survivors [13, 24]. CCSs may apply these strategies because they perceive that cancer has positively influenced them and led to personal growth (post-traumatic growth has been reported in CCSs) or conversely [36], to compensate for negative consequences of the cancer, such as feelings of uncertainty and health concerns [37–39]. Other strategies relevant to post-traumatic growth were evident in “meaning-making” in which CCSs reported appreciating their life, health, and family more. Through “positive appraisal”, CCSs also reported benefit finding and an awareness that there are others who are worse off than themselves. CCSs also reported using strategies to find a sense of normality. All these strategies are relevant to the theory of cognitive adaption which states that individuals who experience a threatening event not even such as cancer may adapt to their cancer experience and new reality by searching for meaning in their illness experience, by attempting to regain mastery over cancer and their life, and through efforts to restore their self-esteem [40].

Peers, and to a greater extent families, have been found to be important sources of emotional and practical support for young adult survivors of cancer [41, 42], and higher perceived social support has also been associated with post-traumatic growth [36]. This echoes our findings of the perceived importance of seeking and receiving social support from these groups, as well as from healthcare professionals within the cancer care team. Many CCSs also specifically mentioned valuing the close links they had with their cancer care team and feeling cared for. Feelings of being attached to the care team and familiar with the paediatric clinic have been identified as an important barrier to older CCSs transitioning to adult LTFU care [43, 44]. However, the possession of self-management skills could improve readiness to transition [43].

In attempts to create a healthy environment, our CCSs reported actively acquiring information, materials. and resources to aid self-management to a greater extent than adult survivors [13]. This may be because all of the CCSs described long-established relationships with their healthcare professionals and most were in LTFU care where information provision about late-effects and healthy lifestyle is an important focus. Additionally, several CCSs reported seeking knowledge about their treatment and late-effects; these survivors were very young when diagnosed and wanted to increase their understanding of their cancer history and its potential implications. Although better understanding of potential long-term consequences of cancer may be advantageous in that it could lead survivors to adopt risk-reducing behaviours, it may also have negative effects. Howard (2016) found that CCSs who talked about seeking information, and who were more proactive in their healthcare, also framed their health as being compromised with worries about potential late-effects [45]. In our study, many CCSs reported the use of strategies to avoid thinking about the potentially negative consequences of the cancer and its treatment in order to protect their psychological health.

Some strategies that are very common in adult cancer survivors (e.g. “proactive problem solving”, “acceptance”, “conserving physical energy”, and “self-sustaining”) [13] were reported far less by CCSs. In addition, almost 20% of the specific self-management strategies from the adult cancer work were not supported in the data of CCSs (e.g. *treating illness as a project*, *drawing on spiritual resources*, *focusing on getting back to work*). This is likely to be due to the differences in participant demographics (age and life stage), clinical management and functional limitations associated with the cancers under study, and time elapsed since diagnosis/treatment. Therefore, although the self-management typologies provide a useful and important starting point, and many self-management strategies will be apparent across different cancer populations, some will differ. This highlights the need to undertake empirical research within the specific patient group of interest and employ inductive analysis alongside deductive analysis, particularly if the findings are to be used to inform intervention development.

It is important to note that higher frequencies of reported use do not necessarily indicate those strategies that are the most important to CCSs or, indeed, those that survivors found most helpful or effective [46]. For instance, “activity-based coping”, “conserving physical energy”, and “conserving emotional energy” were less commonly reported, but for CCSs who did report these, these seemed to be significant for their self-management.

As well as identifying strategies that have been highlighted in previous studies (as described above), we were able to identify a range of strategies which have not been well-reported in CCSs. For example, participants reported cognitive and behavioural strategies of conserving emotional energy, activity-based coping, behavioural avoidance, adoption of health promoting behaviours (other than diet and exercise), use of goal and action setting, approaches to managing others, use of proactive problem solving, use of reasoned decision-making regarding health behaviours, and engaging in self-monitoring. These findings offer an insight into the practical approaches used by CCSs to manage their health and well-being. Moreover, many of these strategies are associated with behaviour change techniques (e.g. problem solving, goal setting, action planning, self-monitoring of behaviour, consideration of the pros and cons of the behaviour), which are the “active ingredients” of behaviour change interventions and, indeed, self-management interventions [47]. This is encouraging and suggests that these particular behaviour change techniques may be acceptable in this population, which is clearly important for intervention development. The range of specific self-management strategies reported by the study participants ranged from 20 up to 70. This highlights that self-management is individual to the survivor and reiterates that support given for self-management should be tailored to the needs and preferences of the patient [12], which is also key for intervention development [48].

The findings also indicate—for the first time—that survivors use strategies associated with behaviour change particularly in relation to physical activity and diet, both of which are reported to be problem areas for CCSs. There is a paucity of research regarding why survivors struggle to maintain positive health behaviours [48], and our research identifies some strategies which could warrant further investigation in relation to specific health behaviours.

A significant challenge for care of CCSs internationally is how to ensure effective transition to long-term follow-up [49]. During this transition, typically the survivor will start to have less frequent contact with their clinical team (a team to which, as demonstrated here, they are often very attached). This is compounded by the fact that the survivor is getting older and parental influence over their health is waning. It is therefore encouraging that so many CCSs in this study recognised that the onus lay with them to take care of their health and well-being, and that they reported engaging with such a variety of self-management approaches.

A strength of this study was its qualitative approach which enabled survivors to talk freely. We had a good response to the study in a patient group well-known for being hard to engage with research [50]. Through the use of content analysis, we were able to provide a snapshot of the extremely varied range of psychological, social, and behavioural self-management strategies used by CCSs, many of which have not been previously reported in this population. We focused on active participation in self-management, in line with the NCSI definition of self-management [12]. This meant that we, therefore, did not address negative self-management strategies (e.g. use of smoking to relieve stress). Further work would be valuable to better understand these more negative approaches.

In terms of limitations, CCSs were recruited from a single clinical site. However, this is the principal treatment centre for whole of the Northern Region of England and provides similar services to principal treatment centres in other regions of the UK. Participants were in follow-up care and CCSs who have been discharged, or who choose not attend follow-up, may report different strategies and potentially be less engaged with their healthcare [51]. The study was presented to potential participants as being about looking after their health; it is possible that this may have resulted in participation of survivors who were particularly interested in this. Although we did not sample for specific late-effects, many CCSs reported a range of issues such as anxiety, depression, diabetes, fatigue, physical limitations, and an increased risk for future disease (e.g. cardiovascular problems, second cancers).

### Implications

In the short term, these findings may help make health professionals involved in the care of CCSs more aware of survivors’ willingness to engage in self-management. They may also indicate strategies that professionals could suggest to their patients. Interventions to encourage self-management are being developed for a range of adult cancers [16]. To inform such interventions for CCSs, it is essential to understand their views and needs and to develop a detailed understanding of why they behave in the way they do [52]. This information is essential to underpin the systematic development of an evidence-based intervention [53], but is often lacking in interventions for CCSs. This study may, therefore, be considered the first step in the development of an evidence- and theory-based self-management intervention for this survivor population. Moreover, some aspects of the findings relate to theories which previous literature support as potentially effective in achieving health behaviour change [54], and therefore could be relevant to improving self-management behaviours in CCSs (e.g. social cognitive theory, self-regulation theory, and self-determination theory). The aforementioned theories can help to identify associated behaviour change techniques that could be valuable and acceptable in this population if incorporated in a future intervention.

## Conclusions

This study has, for this first time, identified the many and diverse strategies used by young adult CCSs to manage the challenges of living with and beyond cancer. The findings may inform the development of interventions to encourage and facilitate self-management tailored specifically for this growing population.

## Electronic supplementary material

ESM 1 (DOCX 82.0 kb)
