# Supplementary material for: Identifying and exploring the self-management strategies used by childhood cancer survivors
Source: J Cancer Surviv. 2020 Nov 6;15(2):344–57. doi: 10.1007/s11764-020-00935-2 (PMC7966631; doi:10.1007/s11764-020-00935-2)
Supplement: Supplementary file 1 — (DOCX 82.0 kb) [file 11764_2020_935_MOESM1_ESM.docx]

**Supplementary File 1: Content codes, definitions and examples for strategy types and strategies**

**Article title:** Identifying and exploring the self-management strategies used by childhood cancer survivors

**Journal name:** Journal of Cancer Survivorship

**Author names:** Morven C. Brown*, Anna Haste, Vera Araújo-Soares, Roderick Skinner, Linda Sharp

**Affiliation and email address of corresponding author:** Dr Morven Brown, Population Health Sciences Institute, Newcastle University Centre for Cancer, Newcastle University, Sir James Spence Institute, Royal Victoria Infirmary, Newcastle upon Tyne, NE1 4LP. Email: morven.brown@newcastle.ac.uk

**Supplementary file 1: Content codes, definitions and examples for strategy types and strategies**

| Content codes for strategy types *Specific strategies* | Definition for strategy type | N | % | Illustrative quotes |
| --- | --- | --- | --- | --- |
| 1. Acceptance | Accepting functional, lifestyle and social changes following cancer and its treatment | **8** | **33** |  |
| *Accepting cancer and its consequences* |  | 7 | 29 | 013 Matthew: My health is going to deteriorate again because of what I had and because of the treatment, it was quite severe treatment and I think you have to have that realism in there, that something will change at some point, even if it’s in another 20 years times.  007 Gareth: I’m still not as strong as I could be or as fast or have as much stamina as I would like to and probably would have if I didn’t have the treatment but I’m still at a good level for what I was in.  024 Daisy: I think that what, over the few years, I've been coming to terms with is well this is never going away and life will never be what it was before. |
| *Accepting new health behaviours* |  | 1 | 4 | 003 Chris: I’m on penicillin and I’m on Metformin for diabetes, so it’s 3 pills twice a day, it’s nothing. |
| *Accepting social difficulties* |  | 1 | 4 | 024 Daisy: Her life went one direction, mine went another and I thought it was me putting the effort into the friendship and I thought, ‘Do you know what? I'm not getting the enjoyment out of seeing this person, I'm not getting anything reciprocated, so fine’. |
| 1. Activity-based coping | Use or uptake of hobbies or activities to manage one’s emotional well-being | **9** | **38** |  |
| *Pursuing an existing hobby/activity* |  | **3** | **13** | 002 Brian: That’s all I did I played Saturday, Sunday before the treatment that’s all I used to do was football and watching it was… I’d watch the footy lads on a Sunday with the team I used to play for its hard like and I just couldn’t stand watching again. I hate watching football like not knowing you can’t play was like …it was mentally it was I’d already gotten the weight off so, it was like I cannae not play.  003 Chris: It’s always been like that because it ties in to when I was a kid. What did I do on the ward, played with my computers all the time. It’s my, it’s my little blanket and now it’s just my bigger blanket that takes up half my room. It’s… the computer’s the blanket, the computer’s the constant it’ll always be here you know what I mean, it’ll never question me, you know, it’s always the constant, it’ll always be the constant.  010 Joanne: I’ve always painted since I was little but when the cancer treatment came on I’d stopped for a while I just didn’t have the inspiration I guess…erm a few months ago I’d eventually started drawing again and I’m back into painting now. |
| *Taking up a new hobby/activity* |  | **7** | **29** | 001 Alice: I’ve took up like different hobbies, like crochet [laughs]…People laugh, they’re like ‘Alice you’re 32 why on earth would you want to crochet?’ I do, I’m not in a bad place really, I’m fine. I just work a lot.  009 Imogen: The team I play for and everyone there is just lovely. I mean, they completely understand.  014 Nick: I’m a Scout leader so I’m out every Monday night with five to eight-year olds. I love that. It gets me motivated. It gets me out of the house. It gets me exercised. It’s lovely. It’s energetic. You enjoy it. You love working with the kids. |
| 1. Adopting a healthy lifestyle | Adopting generic health behaviours to boost one’s general physical and/or emotional well-being | **24** | **100** |  |
| *Adopting a healthy diet* |  | 16 | 67 | 016 Pia: I’ve started eating healthily and not eating as much crap like takeaways and stuff like that. I have started to try and eat a bit more healthily in that way.  008 Hugh: I made sure I had a breakfast this morning and things like that you know, breakfast is the most important meal of the day according to everyone so yeah. |
| *Avoiding negative health behaviours** |  | 15 | 63 | 006 Freya: it’s also kind something that goes without saying that if you’ve had a disease like that you probably shouldn’t be smoking that’s not the best idea.  022 Beth: I’ve obviously stopped going on sunbeds and going out in the sun with no cream on. |
| *Being physically active in everyday life** |  | 9 | 38 | 007 Gareth: Well, my job helps cos I’m outside constantly I’m always moving I’m never not doing nothing.  016 Pia: I don’t sit down all the time. I am active but not active like gym-wise and running and stuff. |
| *Ensuring personal hygiene** |  | 3 | 13 | 022 Nick: Like, part of that being healthy is good appearance, washing your hair, washing under, like, your fingernails and stuff like that, and knowing how to wash your hands properly when you’ve, like, been out in the garden digging or been to the toilet or things like that. |
| *Exercising* |  | 18 | 75 | 012 Laila: I’ve been more active by going to the gym and joining loads of sports that I like doing but I never really did them but now I’m like I might as well actually go and join and do them.  017 Quinn: There were times when I’ve done exercise in the house and stuff like that – lifting weights and push-ups and sit ups and generally keeping in shape.  024 Daisy: I exercise every day. I take the dog for a walk every single day. I don't drive as well so during the day, if I need to nip to get a pint of milk or whatever, I'll walk to get it. |
| *Drinking enough water** |  | 3 | 13 | 022 Beth: I drink a lot of water, I probably drink a couple of litres of water a day. |
| *Meditating* |  | 1 | 4 | 010 Joanne: Erm I do try and meditate but it doesn’t work as much on me. |
| *Reducing negative health behaviours* |  | 14 | 58 | 014 Nick: I cut sugar out. I don’t have sugar in my tea anymore. I used to have a little bit of sugar in my tea.  016 Pia: Drinking, I don’t really drink as much now, but when I first turned 18, like when you first turn 18 you want to go out and have a drink, don’t you? I did used to drink a lot then.  022 Beth: I’ve got to admit, if I go out and have a drink, I do have a few cheeky tabs because I used to be a smoker and I quit once I was diagnosed. |
| *Taking medication** |  | 17 | 71 | 018 Rosie: I take painkillers for the pain that I get. I’ve suffered with pain ever since I came out of hospital, in my legs. Over the past few months of being put on anti-depressants. |
| *Taking vitamins and minerals** |  | 2 | 8 | 008 Hugh: I’ve just started taking vitamin tablets.  024 Daisy: I'm on calcium because they were worried about my bones. |
| *Sleeping well** |  | 1 | 4 | 008 Hugh: So yeah, even like sleeping well and doing exercise. |
| 1. Behavioural avoidance | Behavioural strategies which minimise one’s contact with threats to one’s physical and/or emotional well-being | **8** | **33** |  |
| *Avoiding activities that may cause harm* |  | 3 | 13 | 005 Ellen: Getting a tattoo, I don’t know if that’s just me or the fact that I’ve had a blood disorder - why would I go and get a tattoo?...Why would I have something like that done that’s totally unnecessary, when I don’t even know if it would have any consequences but why take the risk?  022 Beth: I would rather be able to go to the gym and be able to do more activities, but I just end up in pain, so it’s not worth it, for me. |
| *Avoiding contact with others for possible infection* |  | 1 | 4 | 005 Ellen: I do feel like I get coughs and colds quite easily though so just stay away from them. |
| *Avoiding situations that may cause harm** |  | 4 | 17 | 009 Imogen: It also depends on the environmental conditions as well, because obviously if it's wet I don’t like walking on the sticks because you know I could slip and hurt myself.  014 Jason: I avoid it as much as I can. Like I’m at the bus stop and there are people smoking at the bus stop who, “Excuse me, will you go and stand outside the bus stop and smoke?” Because I’ve had cancer. Or, if there are people standing in the doorway and I need to be in, well I go, “Excuse me, can you just stand to one side while I go in? And then you can come back there and have a smoke and finish your tab or your ciggie.” |
| *Avoiding uncomfortable social encounters* |  | 3 | 13 | 007 Gareth: I really only learned to swim just after my line got taken out so I was like one of the last one’s in my year to actually learn how to swim. So it was a bit embarrassing for me in that sense cos I didn’t really tell people like if they said ‘can you swim?’ I avoided that question as much as I could (laughs).  021 Abigail: If I was running late for college and I didn’t get a chance to do my make-up, I’d like to try and keep myself to myself, not go anywhere that I might recognise people. |
| 1. Cognitive avoidance | Strategies involving the avoidance of thoughts concerning the negative consequences of cancer and its treatment | **13** | **54** |  |
| *Avoiding finding out too much* |  | 3 | 13 | 013 Matthew: I’ve no intention of searching the internet and digging around and finding out more what could go wrong, and I don’t feel the need to ask [consultant] any more than he already mentions. |
| *Avoiding thoughts about cancer and its consequences* |  | 8 | 33 | 014 Nick: You’ve still got that on your mind but not focusing on that. You put it to one side and focus on what matters. |
| *Dealing with (in)fertility at the right time** |  | 4 | 17 | 003 Chris: That’s not something I want to talk about with em…with anyone, not yet, not ‘til it matters…Emm not ‘til it matters in my head. Cos it matters but they didn’t say I was infertile, they just said it was a low sperm count.  021 Abigail: I’ve got quite a serious idea of when I, it’s quite a bold statement, but when I think it’s right to have one [a child]…but with the cancer possibly affecting erm being able to have one myself, I think that’s probably, it’s an issue I’m going to cross but when the time is right. |
| *Distracting oneself by keeping busy* |  | 3 | 13 | 003 Chris: It only ever comes when I’m er under stimulated or alone em normally at night when I’m trying to sleep because I’m switching off. I fill my days with either… trying to go out and do some kind of voluntary work because I’m currently unemployed… Em or I sit at home and I just lock myself in my room and play games, watch, watch movies and shit, just stuff to keep me, keep me busy, you know what I mean?  012 Laila: Just keeping myself busy and not thinking about stuff because when you just sit and you have nothing to do and you just think it goes downhill. |
| 1. Conserving emotional energy | Strategies which enable one to conserve emotional energy in order to better self-manage one’s condition | **15** | **63** |  |
| *Caring less about what others think §* | Not found in data |  |  |  |
| *Having time to yourself** |  | 6 | 25 | 002 Brian: I like that suppose running’s not too bad cos it gets you out and it gets you like you can go for a run it gives you time to yourself as well cos you de need time to yourself.  018 Rosie: I won’t be as pissed off, because I’ve had that five minutes break where I've concentrated on myself for five minutes.  004 Ella: I think taking some time out sometimes, if I’m studying a bit, and needing a bit of a break, I think that can, I think you do the world of good sometimes as well and just you sort of refresh your mind a bit which I also think is quite important. |
| *Letting emotions out** |  | 3 | 13 | 018 Rosie: Like, how I deal with it?... Sit and cry [laughter]. Honestly… I am sometimes just sat there crying. My boyfriend will come over to me, and he’s like, “Why are you crying?” Because I am.  005 Ellen: I like to sort of I’ve got like an app on my phone that’s called coloured note and you can its sort of like post it notes so you can just post it note and I’ll often if somethings really, really griped me I’ll often go in and do a bit of a diary insert just write it all down just eurgh just eurgh it out and then just put it away and I can just eurgh it out on there, be frustrated be annoyed, get it all out and then just leave and then a week or two later just delete it. |
| *Minimising stress* |  | 4 | 17 | 010 Joanne: I think the main aftermath is just trying to just not get as stressed out. |
| *Switching off** |  | 7 | 29 | 008 Hugh: Cos even when I’m like in the car I always like to listen to music it’s a way of switching off.  003 Chris: I’ve got a specific thought process that shuts me down. If that thought process is interrupted by a bang or a noise or anything really that’s loud enough to break through me semi-conscious kind of sleep state, then I won’t be able to sleep for another hour.  013 Matthew: Just the escapisms, the music, books, that sort of thing. |
| *Using sleep** |  | 3 | 13 | 013 Matthew: At university …if I was having a bit of a difficult time, I would have an afternoon nap or something, certainly not in the real world now, just sometimes. Some of the difficult times at uni, I did just end up having lots of afternoon naps, just waking a few hours, got it out the way.  004 Ella: I frequently have naps during the day as well which isn’t great. I do try to keep them to a minimum, but I think sometimes getting my head down for an hour, it just makes me feel so much better.  014 Nick: I listen to music, quiet, calm music. Or sometimes I don’t have anything on, I just try and nod off and go to sleep. Stuff like that. |
| 1. Conserving physical energy | Strategies which enable one to conserve physical energy in order to better self-manage one’s condition | **5** | **21** |  |
| *Reducing activities* |  | 2 | 8 | 024 Daisy: I was working eight until six at a cafe, on my feet all day and I just got on with it and it was fine. Now, if I walk the dog for an hour, I need to sit down after that. |
| *Reducing workload §* | Not found in data |  |  |  |
| *Taking a break* |  | 4 | 17 | 004 Ella: I have to sort of make sure before I go, I do have a lot of time out sort of just because I know that later on, if I’ve had a tiring morning, that it could have adverse effects later on. |
| 1. Creating a healthy environment | Attempts to create an environment which enables effective self-management | **24** | **100** |  |
| *Acquiring knowledge about cancer, treatment and late effects and available support (was ‘acquiring knowledge about condition and available support in Dunne 2017)* |  | 11 | 46 | 008 Hugh: it was always part of my life like coming for follow ups or I was always like ah I’ve got to go to the hospital but it’s not since the last good few years when [consultant] left and things like that I thought well am I actually coming here? Then I started to think well why and what happened and obviously I started to ask me mam more and me grandparents more.  003 Chris: obviously as you get older and we have the internet you look stuff up so you look you know first time I looked up exactly what had happened.  001 Alice: Yeah I’m very, I’m quite clued up since that I’ve, since like Googled the kind of medication, not Googled, but you know what I mean I’ve looked up the medication I’ve had, and consequences. |
| *Attending follow-up and screening appointments** |  | 20 | 83 | 011 Keith: I see [consultant] once a year, to see… an MOT basically, just to check all the functions are working and alright, you know?  013 Matthew: I’ve got that age where I’m starting to get some of the after effects of the chemotherapy and the radiation therapy and [consultant] has always been doing tests for lung function, heart issues that you mentioned before, joints, bone density, all that sort of stuff. |
| *Collecting materials to aid self-management* |  | 14 | 58 | 007 Gareth: Erm well, I got given that little info pack once I got admitted to the follow-up properly and so everything is in there if I just need to check something I always check that really.  008 Hugh: they [consultant] gave me a sheet of obviously what operations I had, erm obviously the drugs I had and it even had the amount of drugs I had and obviously they were saying well this one here is obviously…obviously always check your heart for cos it could affect this, could affect that.  014 Nick: I got information about it [looking after health] and I got leaflets from my social worker, from CLIC Sargent. |
| *Ensuring reliability of health information on the internet ** |  | 5 | 21 | 003 Chris: Don’t Google the side effects, ask a medical professional because Google is the devil when it comes to stuff like this. Em you know no matter what happens right, you could have a cold but the internet will say you’ve got cancer, [laughs] I swear to God it’s sooo stupid. ‘I’ve got a bad arm, oh it’s falling off because of cancer’, ‘no, no, internet no it’s not’ and this is not like the NHS websites. |
| *Learning self-management skills* |  | 2 | 8% | 021 Abigail: It’s like to do with my injections, many times she’s [nurse] had to come to the house cos of the two new models I had, and she would just sit in the conservatory for hours on end, just explaining how to use this thing.  022 Beth: I would actually be looking into things like stress management, coping mechanism, I was looking at that anyway, so I’ve tried most of them and it’s trial and error, what works for some people and doesn’t work for others. |
| *Obtaining resources to aid self-management** |  | 10 | 42 | 006 Freya: I’ve got a gym membership and I use that and I do classes and stuff.  008 Hugh: I’ve just obviously went to [sports shop] the other day and got myself some shorts and tops and I was like right I’m going to do it now look I’ve got no excuse the only thing I’m missing obviously is a holder for me phone or me iPod so I can put that in and then put it in me ears and I can run.  007 Gareth: I mean I’ve got cookbooks and that so I’d always look through them, I’d always look on-line just to see like quick options and stuff to see what the crack is. |
| *Relationship-building with health practitioner §* | Not found in data |  |  |  |
| *Utilising skills for independent living** |  | 9 | 38 | 007 Gareth: I still know what I can do and how much I can do it cos obviously I’m paying for it now erm then I know how much I can and what I can do and then budget it that way. Erm I’ve got my car to think about as well cos obviously that’s my transport everywhere so then its fuel for that I’ve got to budget massively.  015 Oonagh: I sort my own tablets out. I don’t like people touching them. I have to have them a certain way. I always take a pack with me even if I go out just in case I stay at my friend’s house.  023 Charlie: Because I think I can look after myself. I can use the oven and stuff. |
| *Valuing and respecting relationship with cancer care team** |  | 12 | 50 | 002 Brian: When you come in it is nice to see everyone I suppose cos I do talk. I do talk to everyone a lot.  008 Hugh: I always feel valued and cared for don’t I? So I feel well I have to go. I mean I wouldn’t be here today if it wasn’t for their hard work and you know what I mean, for them so I wouldn’t … he didn’t let me down so can I really afford to let them down?  009 Imogen: I mean even still to this day, two years on, if I'm not feeling well or I get some back pains we’ll still phone the hospital, just let them know what’s happening. |
| 1. Goal and action setting | Use of planning or goal-setting self-management strategies | **19** |  |  |
| *Coping planning** |  | 4 | 17 | 001 Alice: Every time I wanted one I’d say to myself ‘you don’t need to smoke’, ‘I’m not a smoker’, used to keep myself busy which is probably more effective than the medication because you don’t have all the side effects.  010 Joanne: If I do feel like really peckish or whenever I have like a munchy day I’ll just go and walk with the dogs I’ll take them for a long walk just to keep my mind off it. |
| *Planning daily activities* |  | 11 | 46 | 002 Brian: I do try and plan it was like 3 runs before but then I say I used try I want to go to the gym 3 times a week as well but it’s hard to like do it all if I could get up early enough in the morning I do think I would be able to do it but I can’t I just can’t get up like I do try and plan it all out but I it just never works me plans never go to…  005 Ellen: I’ve put like an alarm on my phone and I’ve made it a different time of the day so it was like – right the alarms going to go off when you get to work and when you go to put your food in the fridge then you’re going to take them because that’s when you’re alarms going to go off its going to tell you to do it now, you’re going to do it.  006 Freya: Erm at the moment because I’m a student I have a bit more flexible time it’s easy for me cos I can design my day around when I want to go to the gym and stuff.  008 Hugh: when I go to work I do night shift as well and they can be quite brutal night shifts 12 hours especially, so erm even when I go on night shift I always take pasta and tuna and usually things like that I used to take ready meals. |
| *Priority-based planning §* | Not found in data |  |  |  |
| Setting future goals |  | 13 | 54 | 005 Ellen: I do wanna lose a little bit of weight because I am slightly more than I should be. I think I’ve 8lbs to lose to get to where I want to be.  007 Gareth: I just try to push meself so if I’ve got to one stage one year I want to get to the next stage next year just like setting goals really. |
| *Setting up facilitating conditions* |  | 11 | 46 | 003 Chris: it varies it does vary em I have them in locations in the house that I tend to frequent often so I will see them and then I’ll check, do a mental check, have I had it yet, no.  007 Gareth: Yeah so it’s like I’ll finish work, I’ll have prepared something like I don’t know the day before or before I go out so I can literally just turn it on so its busy cooking so then I can just sort everything out and then eat, either go out and come back have another snack and then sleep.  014 Nick: Writing it down so you don’t forget. |
| 1. Managing others | Active attempts to effectively manage one’s social relationships following cancer treatment | **12** | **50** |  |
| *Avoidance of negative relationships** |  | 3 | 13 | 015 Oonagh: Definitely negative people, and people who like to put you down. I try to keep away from those types of people ... |
| *Being assertive in social encounters* |  | 4 | 17 | 014 Nick: Like I’m at the bus stop and there are people smoking at the bus stop who, “Excuse me, will you go and stand outside the bus stop and smoke?” Because I’ve had cancer. Or, if there are people standing in the doorway and I need to be in, well I go, ‘Excuse me, can you just stand to one side while I go in? And then you can come back there and have a smoke and finish your tab or your ciggie’. |
| *Being open with others about cancer and its consequences* |  | 4 | 17 | 009 Imogen: I mean I'm not afraid to talk about anything. If anyone asks me any questions, I'm like, ‘Don’t worry about it. I’ll happily tell you anything you want to know.’ It's not something I'm going to hide. |
| *Keeping others happy* |  | 2 | 8 | 008 Hugh: If I’ve upset someone if will really like ah or even if I’ve been out on a night and I fell out with someone it will still affect me like the day after. I can’t eat cos I feel that bad but I’m kinda like one of those people that’ll if I upset someone or things like that I’ll always like to stay if I… the one that makes, like brings people back together or fixes things sort of thing. |
| *Protecting others from harm* |  | 1 | 4 | 013 Matthew: So most of the other stuff just affects me, so obviously I can reconcile a lot with myself, but this potentially could affect someone else. |
| 1. Meaning-making | Interpreting cancer and its consequences in the broader context of life as a whole | **18** | **75** |  |
| *Appreciating health more* |  | 12 | 50 | 006 Freya: I think maybe I’m more health conscious because of maybe what I’ve been through so like I know what it’s like to not be in control of your own body kinda so maybe that might be something that kinda motivates me a bit more than say anyone.  022 Beth: I’ve just had my smear test last week and things like that, before that I might have thought, ‘oh it won’t happen to me, it’s not really important that I go for it now’ but now I think I’ve actually definitely got to go because these things can happen and we’re not invincible. |
| *Appreciating life more* |  | 10 | 42 | 003 Chris: I mean I’m not a religious man but I’ve always said if I was to believe in any higher power it would be that specific event in my life that would make me believe that.. I’m here for some particular reason or something.  013 Matthew: You think well that could have been the end, but it wasn’t, so this is extra bonus time, sort of thing, why waste it being all miserable and doing nothing when I could be experiencing things and seeing friends and doing stuff. |
| *Appreciating support* |  | 7 | 30 | 008 Hugh: Me grandad obviously brought me in I might not be here today so it’s all down to him. As you get older you properly realise if me mam or me grandparents didn’t do that, imagine, do you know what I mean? So I owe a lot to them.  014 Nick: I was much happier with the treatment that I had, the support. I couldn’t have asked for better. The consultant, he’s one of the best.  011 Keith: There’s a lot of people put blood, sweat and tears into trying to keep me the way I... to keep me alive, so I’m not going to just forget about it and just go on living my life like a normal 26-year-old which are probably partying and smoking. |
| *Appreciating the importance of family* |  | 7 | 30 | 007 Gareth: I mean I’ve got all the support I need really I’ve got my family like they’ve always been there. I know they always will be they’re like the reason I do what I do. |
| *Appreciating the severity of one’s cancer history** |  | 5 | 21 | 011 Keith: I think there was a big effect that when you’re a kid you don’t understand, like, as I said earlier, what you’re going through, but as you get older, you start realising how much... what I went through, and how much it could have been a lot more serious. |
| *Becoming more altruistic* |  | 6 | 25 | 011 Keith: I’m not a new person, but I’m a person that understands. |
| *Changing one’s image §* | Not found in data |  |  |  |
| *Finding meaning in work* |  | 2 | 8 | 003 Chris: I think that plays on your mind as well when you’re not, you’re not part of a routine you’re not part of anything. That can always lead to you know mental erosion as well if you’re not engaging enough. |
| *Taking every day as it comes** |  | 7 | 30 | 001 Alice: So I’m very much like live each day as it comes, do what you want to do and try not to worry too much but not be so ridiculous that you’re putting your life at risk all the time.  010 Joanne: Erm with me having so many tablets during chemo I didn’t like the thought of going back tablets for depression so I thought I could manage by myself and just take it day by day, step by step like I did with the chemo. |
| *Wanting to give something back** |  | 3 | 13 | 014 Nick: Like me and a few others, we’ve done charity events. I’ve stood up in front of my high school. I’ve stood up in front of my college. I’ve stood in front of primary schools and I’ve delivered assemblies about cancer, what the side-effects are and how you can get it. |
| 1. Positive appraisal | Focusing on positive aspects of one’s immediate situation | **13** | **54** |  |
| *Benefit finding* |  | 7 | 29 | 001 Alice: Yes I think I grew up quicker, yes I think it makes me more aware of people around me and looking after me and em I’ve got knowledge that I maybes wouldn’t have had had I not have been through that.  016 Pia: It sort of brought me out of my shell a bit more. I used to be shy and I never used to speak to anybody, really. But now, I’ll speak to anybody, really. Like if I go round to my local pub or something, I don’t mind sitting and having a bit of conversation with somebody. Whereas, before I had the cancer I wouldn’t even say ‘boo’ to anybody. |
| *Downward comparison* |  | 6 | 25 | 003 Chris: If I’d got it as a teenager I’d have been twice as bad as I am now I think. Because I’d have been self-aware, I’d have carried it on, I’d have gone bald in my teens which is going to be an issue in itself, I’d have been infertile, well a low sperm count in my teens, and all the things that matter would have just got thrown out of the window. And I always hold a huge amount of respect to all the kids who get it when they’re teenagers, especially the women. Because I think it’s it would be much harder for a girl going bald than it is for a lad.  009 Imogen: there are people in worse mobility situations. I mean I play wheelchair basketball and there are some people that you know can't even leave their wheelchair, so in a way like that I'm grateful for what I've got.  022 Beth: I’m so grateful I didn’t have to have horrible treatments like chemo and radio because obviously I’ve got a lot of friends from the Teenage Cancer Trust and I know that it is horrific in the long-term. |
| *Reinterpreting negative consequences* |  | 1 | 4 | 005 Ellen: But you could have rubbish hair at any point in life, it might just be me, might be nothing to do with anything else, it might just be me. |
| 1. Proactive problem solving | Active attempts to solve problems in-the-moment arising from the consequences of cancer and its treatment | **10** | **42** |  |
| *Acting to prevent further complications* |  | 1 | 4 | 005 Ellen: just like when there’s colds going round the school and things like I just make sure I’m washing my hands plenty and things like that just trying to be a bit more on the ball making sure I’ve got my flu jab. |
| *Adaptive approaches to ongoing physical consequences of cancer and its treatment* |  | 5 | 21 | 006 Freya: I started swimming cos it’s kinda less impact on your body.  008 Hugh: I used to always last the full match I used to be a defender anyway so it was quite… I’m not very good with me feet do you know what I mean so I used to say ‘ah I’ll just defend’, things like that.  009 Imogen: I still use my Zimmer frame in the house but I mean that’s just because it's quicker. |
| 1. Reasoned decision-making | Objective decision-making strategies relating to survivor self-management | **24** | **100** |  |
| *Considering benefits of positive health behaviours** |  | 18 | 75 | 005 Ellen: I always do feel better after I’ve done something, done the exercise but then again I always feel better after I’ve had a massive piece of cake but it’s true though if you eat too much junk you do feel naff and I always do feel better after I’ve done the exercise.  013 Matthew: If you come out the gym and you feel like you can function better, you’re feeling more positive and awake and more active, that sort of thing. So, you think well there’s definitely a benefit to this, so keep going. |
| *Considering pros and cons of self-management* |  | 12 | 50 | 005 Ellen: I know I’ve got the energy to be able to do something on a Tuesday where I think if I stepped it up too much I would just end up tired, too tired.  006 Freya: I suppose you do have to erm pay for the prescriptions every time you get them and I’m going to have to take them for the rest of my life so obviously it would rack up over the years. I suppose it is an expense that I will have to take on.  015 Oonagh: Also, with my epilepsy which connects to that and the tablets I am taking, it always concerns me that if I have a child then it isn’t going to be healthy because of my tablets which would mean I would have to come off my tablets and face whatever happens or stay on them and cause other things. |
| *Evaluating effectiveness of self-management* |  | 8 | 33 | 003 Chris: I’ve had the same optimal weight I’ve had for 10 year so I think I’m doing well on that side of things. I mean I don’t hammer the gym, but I don’t consider myself to be super unhealthy.  017 Quinn: I do eat fairly healthily but obviously because of the amount of alcohol I drink it sort of goes out of the window a little bit – it sort of cancels it out a little bit. I make salads and good food and then you decide drinking… well over the recommended amount, you’re no longer effectively healthy. |
| *Thinking objectively about negative health behaviours* |  | 19 | 79 | 017 Quinn: I presume that I can’t probably continue to drink this much otherwise I’ll have problems in later life  018 Rosie: Stop smoking. That would help. Not just health wise, but also financially.  019 Scott: if you were eating the wrong foods and like didn’t watch your weight and things like you’d probably be just stuck in the house all day, you’d get bored you’d get agitated and just probably get depressed after a while. |
| *Thinking objectively about negative thoughts and emotions* |  | 7 | 29 | 008 Hugh: I always think I knock meself when I shouldn’t and I need to feel comfortable with myself I think I can do things and I can talk to people and I’ve got I don’t know I’ve always had a little bit of something holding me back or doubt or if I’m not good enough or I don’t know what it is and its even people have said it before there’s something that’s holding you back even though I’ve done well to where I’ve got to today so and I do look back and think I have done well but there’s still things that I feel I can work on and …  024 Daisy: I'm trying to get out of that mentality of ‘I need to be this weight’ and focus more on ‘I feel a bit better’. I still struggle with the clothes size thing. My mum is like, "You need to get out of that as well." It's just I'm trying to work on that, that there are bigger things in life. |
| 1. Seeking normality | Active attempts to return to normal living following cancer treatment | **21** | **88** |  |
| *Balancing life with health needs** |  | 13 | 54 | 011 Keith: My mindset is, like, 90% is mainly focused on getting myself fit and healthy, helping myself make sure I can do the stuff I want to do, not restricting myself. The other 10% is just let’s have fun sometimes.  012 Laila: It’s easy and it’s not cos at times like you do want your cheat days and just not do or care about anything but then you do have to also like watch out for stuff and then like you know checking yourself and but then you just feel like you can’t have a normal day like everyone else because you’re always like trying to be on top of your health so it’s a bit of both. |
| *Carrying out tasks to the best of one’s ability** |  | 2 | 8 | 008 Hugh: I always feel when I go into work I can still do it to the best of my ability sometimes I go home and I can be absolutely shattered and knackered. I’ve still done it, do you know what I mean? |
| *Choosing when and to whom to disclose cancer history** |  | 7 | 29 | 007 Gareth: I don’t really tell people that I’ve had it and stuff cos as soon as I do I get tret differently and there’s no point in that when I’m in the clear at the minute so there’s no point in being tret differently for something that I know isn’t here.  013 Matthew: You start thinking it’s going to impact on those relationships, because it’s a difficult thing. People obviously want children and it’s a burden to think, when do I tell people, do I tell people, what stage? |
| *Focusing on doing normal activities §* | Not found in data |  |  |  |
| *Focusing on getting back to work §* | Not found in data |  |  |  |
| *Gaining independence** |  | 6 | **25** | 009: It builds up my strength as well with me being in a wheelchair. In a way it helps with my independence, so as I'm getting stronger it means I can go further in the wheelchair by myself…without having the need for someone to push me.  024 Daisy: So it's been a very gradual process of, I guess learning to be an adult again or gaining independence again. |
| *Maintaining independence §* | Not found in data |  |  |  |
| *Regaining strength** |  | 5 | 21 | 006 Freya: I think you just kind of adjust naturally like, little by little as you get stronger you can do more so I think it’s kind of a process that you kinda just go through like develop. |
| *Returning to normal** |  | 10 | 42 | 005 Ellen: I think it’s just getting back to normality really, just getting back into school and things like that  015 Oonagh: It’s generally getting back to yourself and doing things that you used to before you got diagnosed. |
| *Testing oneself §* | Not found in data |  |  |  |
| *Trying to fit in** |  | 2 | 8 | 015 Oonagh: Seeing people around me look well and making sure that you look similar to them and that what they look like, you look like too. |
| 1. Self-monitoring | Active self-monitoring of one’s health, well-being and ongoing care. | **18** | **75** |  |
| *Knowing your body** |  | 6 | 25 | 013 Matthew: I think to be fair, you can feel your body responding to these things, whether, whether it’s me eating well or it’s because of the treatment, my tolerance to that sort of stuff is lower anyway. So, my body tells me stop drinking or whatever and that’s fine, I’ll do that.  014 Nick: if your body tells you to stop and it’s not coping with the exercise, you stop, have a rest and then do it again. |
| *Monitoring emotions* |  | 3 | 13 | 012 Laila: Hormonal wise as well I think I’ve become more emotional erm like I don’t know I just feel like there’s times where like I wouldn’t be as emotional as I am now. That’s something you can’t really control, so that’s one thing as well. |
| *Monitoring for symptoms of cancer and late-effects*  *(was ‘monitoring symptoms and side effects’ in Dunne 2017)* |  | 4 | 17 | 003 Chris: Keeping an eye on out symptoms you know what I mean. Blotches, er itches, reactions, the general check list [consultant] gives you anyway when you go there, if you have shortness of breath all that stuff, it can be tied into what your treatment’s got.  005 Ellen: I think I’m just more sort of open to the fact that it happens and especially keeping your eyes open for those like secondary things cos, its more likely to get the secondary cancers cos obviously, my system must be done in from all of what I’ve had. |
| *Monitoring general health* |  | 5 | 21 | 002 Brian: Even now like I think even now that probably is in your mind like if I do, there’s time where I put on I can eat I can eat like mad if I don’t exercise, I’ll eat but like if I put on a bit weight I notice more like.  003 Chris: I don’t look at myself in the mirror and think you need to lose weight cos if I did I would do something about it. |
| *Monitoring health behaviours** |  | 11 | 46 | 001 Alice: I keep me little step counter on on my phone every day and when I’m at [work] I can sometimes do up to 28,000 steps in one shift. So I suppose on a Friday, Saturday and Sunday I do my weekly step allowance.  006 Freya: if you do something that might be like not so healthy, I always just make up for it another day or eat healthy another day if I’ve been out for a meal or something.  009 Imogen: I mean sometimes I will be like, ‘Hang on, Imogen, you’ve had three takeaways this week, better have salad.’  017 Quinn: I do look on the back of a bottle of wine now, like, nine or ten units. So there’s nine or ten units in a bottle.  019 Scott: Just probably getting the right mindset to kind of just consciously think that I’m going to start eating right or do a bit more exercise or even keep a journal to say what I’ve eaten. |
| *Recognising one’s own limits** |  | 10 | 42 | 003 Chris: Em I think it’s it’s, there’s certain jobs I couldn’t do at this point, until I’ve developed some kind of coping strategies.  007 Gareth: I always know I can’t push meself too much anymore. I know pretty much that I’ve got a set limit quite early on but I do [sports]. So it’s not stopping me, it’s just I can’t put as much in as I would like to.  012 Laila: with friends like socially like they’d want to do stuff where like for example say go skating or stuff like that I would not be able to go I wouldn’t be able to literally, I know I wouldn’t be able to handle it like where people my age if you fall you’re fine with but I knew if I fell it would have caused great difficulty for me.  019 Scott: not recently but like the first like couple of years after all the treatment finished and that with the radiotherapy, I still got tired really easily so even when I played football with my friends, after about 5 or 10 minutes I just had to take myself away and just sit down for a minute or so. |
| *Monitoring relationship with health professionals §* | Not found in data |  |  |  |
| 1. Self-motivating | Strategies which help to motivate oneself to effectively self-manage | **24** | **100** |  |
| *Being healthy for sake of one’s family** |  | 1 | 4 | 020 Tara: I try and use my family as, oh I can’t think of the word, I can’t think of the word…erm motivation, that’s it, motivation. |
| *Challenging yourself** |  | 6 | 25 | 002 Brian: I try to better meself every time I’m very competitive anyway I think that’s why I do it.  021 Abigail: I think it’s the idea of having something different because going from, I like the change, I like big changes. |
| *Developing confidence and self-efficacy** |  | 3 | 13 | 015 Oonagh: My mum likes to come to my hospital appointments but sometimes she can’t. Normally because I am 21 now I have got enough confidence to do it. |
| *Drawing on spiritual resources §* | Not found in data |  |  |  |
| *Drawing strength from past experiences** |  | 10 | 42 | 008 Hugh: I do look back and think I have done well but there’s still things that I feel I can work on.  013 Matthew: I think it’s made me tougher and if anything, you think well this is pretty bad, there’s not much more that can be worse than what happened the second-time round. You already having dealt with it, it’s, in a strange way, easier to deal with. Other things that happened, obviously that’s how things are changing and stuff but to me they’re just all minor compared to other stuff in the past, it’s just another minor adjustment to make or a bit of an irritation but it will be fine.  015 Oonagh: I do think I look after myself better now than I did a few years ago. Then I didn’t have as much life experience as I do now. Now, I have that life experience and have friends who have gone through stuff it has made me realise that I need to keep going and to keep myself healthy.  016 Pia: It’s just the thought of what I’ve been through and stuff, and trying to make life better, if you know what I mean. |
| *Employing a determined attitude* |  | 11 | 46 | 010 Joanne: The doctors wanted to make sure I was safe enough to go through treatment so he put me on steroids so I was determined on losing them, losing the weight after chemo.  010 Joanne: You just think to yourself no I’m not going to give into my feelings I’m not going to give in to my emotions I’m going to do it, I’m going to feel happier after, and you do.  011 Keith: As long as my mind is set to it, I’ll do it, and I’ve got no other way of thinking about it, to be honest. I feel pretty confident that myself will be... I’ll be pretty healthy within the future coming up as well. |
| *Encouraging oneself* |  | 14 | 58 | 005 Ellen: I forget to take it [medication] and then it will get on my mind. But then I think “go & do it then”, but its.. I’ve got no idea why, no idea why... I think maybe somewhere in the back of my head I think ‘well you’re alright, you’re alright, like it’s alright you’ll be fine’ and the other one’s saying ‘you’ll not be, take your tablets’. So it’s that double nag and I’ll be like ‘I’m definitely doing it’ and I’ll go and do them [medication].  008 Hugh: I used to take ready meals one night I was sick and I thought ‘that’s it [Hugh] stop being lazy and just make something’ so I always used so before I go on nightshift now I always boil some pasta.  011 Keith: Like, a couple of weeks ago, I was feeling dead down, I had headaches and everything, so I couldn’t be bothered. I thought, ah, no, I’ll go to the gym. I had ten minutes in there, I was on the treadmill, and I thought, ah, I could do another five minutes, I could do maybe another... |
| *Focusing on milestones of survivorship* |  | 7 | 29 | 009 Imogen: I feel like no matter what I tried it doesn’t get rid of that anxiety you have before a scan, or even after when you're waiting for results. I've just got to think… I mean I've only got three years left of it until I'm officially declared fully cancer free and discharged from the hospital.  008 Hugh: I got a letter the other day saying I was going from every year to every 2 years now so maybe that’s a good sign to think we’ve been seeing him every year now for 20 odd years now maybe he’s going to be alright obviously we still want to keep an eye on him here and there so do his bloods and things like that.  012 Laila: they did say if there was a chance of it coming back in the first two years after, but it’s been three years now. ‘Touchwood’ - nothing’s happened, so I’m pretty confident. |
| *Interacting with others** |  | 9 | 38 | 004 Ella: I don’t always feel like it’s definitely the thing that I want to do, I just sometimes feel like I should be a bit more social and get out of the house a bit as well.  013 Matthew: Sometimes you think no I don’t want to talk to anyone, I’ll hide in the house. But other times you think well actually, that means I have to talk to someone and you put on an act, almost and by doing that, it actually helps, if that makes sense? |
| *Maintaining a positive outlook* |  | 12 | 50 | 012 Laila: I have a more positive outlook now than I did when I was on treatment. When I was on treatment I thought, ‘It’s never going to end, this treatment’. And I did want it to end, which it did, and I came out positive and I see the light at the end of the tunnel, which most people don’t.  012 Laila: I want that to be there forever cos that’s one main thing that has kept me going is thinking positive.  018 Rosie: It will never leave my mind. It’s just one of those things that will always be there. It’s just something that I've got to live with, in a way. You just got to be positive about life, I suppose. |
| *Not dwelling on the past** |  | 4 | 17 | 011 Keith: I see the cancer as... it’s the past. I’ve got by it. I’ve come out of it.  021 Abigail: Erm, probably quite a major thing, just don’t dwell on what’s happened because if we’re all gonna cry about things that have happened, we’re not gonna be able to get on with things really. |
| *Persevering with healthy behaviours* |  | 7 | 29 | 005 Ellen: Like going to school on a Friday with my excuse of why I can’t do fitness this afternoon but then I always do it and I always come away feeling a lot better for it.  015 Oonagh: When I just started taking them [medication] I wasn’t really that bothered about them. I would just take them here and there but now I have to. I have been taking them for four years now. |
| *Recognising the need for motivation and discipline** |  | 11 | 46 | 001 Alice: I know I can do anything if I put my mind to it but my mind needs to be there and I think again, no excuse, but the job I’m in at the minute I just don’t have that motivation or that kind of oomph to go off and do something for myself.  014 Nick: Sometimes I get up and I go oh, I’m definitely going to the gym, and I look out the window and I say oh, I can’t be bothered to go out in this weather it’s horrible. It shouldn’t be like that. It should be, like, whatever the weather is I’ll go.  016 Pia: I think I’ve just got it [eating more healthily] in my head and I just felt motivated to do it. |
| *Rewarding oneself §* | Not found in data |  |  |  |
| *Taking responsibility for own health** |  | 14 | 58 | 005 Ellen: I think you can’t just sit back and think well I’ve had, you can’t just blame it all on that you can’t blame it all on the cancer, it would be lovely to I’m fat because of the cancer that I had 12, 15, 16 years ago no you’ve got to take some responsibility for what’s going on now as well you can’t just blame it all on the past cos it might not be a helping factor but it’s not all of the problem.  008 Hugh: As I’ve got older now obviously it’s kinda hit us that I have got to look after meself. |
| *Treating illness as a project §* | Not found in data |  |  |  |
| *Wanting to look good** |  | 5 | 24 | 002 Brian: I do it as well cos I like to, you want to look better as well cos I said like when I put on the weight I didn’t look good I looked bad and I couldn’t me self-image is, like it’s quite like, I think it gets you it got me down.  022 Beth: Well I just really want to stay slim and things like that, appearance wise, I guess, it has a big influence and not being able to go to the gym a lot, I do feel like I need to moderate my diet, otherwise I would put on weight and things like that. |
| *Wanting to stay in good health** |  | 16 | 67 | 006 Freya: I’d like to be able to control my own health for as long as I can really.  007 Gareth: Obviously I don’t want to get my disease back and that so I got a bit of like I’m trying to just stop it coming back so I’ll do anything I can just to prevent it.  004 Ella: I think there’s a lot of other things that you wouldn’t be able really to do or to manage if you didn’t manage your health particularly well. |
| 1. Self-sustaining | Strategies which enable one to implement self-management strategies consistently in one’s daily life | **14** | **58** |  |
| *Following health practitioner’s advice* |  | 9 | 38 | 007 Gareth: I don’t drink excessively often cos I know that was one of the things I got told I shouldn’t do.  010 Joanne: Well for one I’m an ex-smoker so when I first mentioned to the doctors, consultants that they told me off they were very firm about smoking.  011 Keith: I have taken his [consultant’s] advice a little bit by changing how heavy I’m lifting. |
| *Incorporating self-management behaviours into daily routine* |  | 8 | 33 | 003 Chris: Yeah it’s just routine. It’s just em the only times I ever waver from the routine is if I go out for the weekend and I forget to take it [medication] because one thing leads to another, but I’ve spoke to [consultant] about that before and he says it’s ok to miss one or two.  006 Freya: I’ve started to do it [exercise] in the past couple of years and I started to get into it and I started to enjoy it and it became part of like my kinda opportunity to do things so I would just say at the moment it’s just something it’s like part of what I do as my free time. |
| *Maintaining medical*  *equipment §* | Not found in data |  |  |  |
| *Customizing dietary practices §* | Not found in data |  |  |  |
| *Keeping busy to avoid negative behaviours** |  | 2 | 8 | 010 Joanne: If I do feel like really peckish or whenever I have like a munchy day I’ll just go and walk with the dogs I’ll take them for a long walk just to keep my mind off it.  016 Pia: Being at work. Because I’m busy and don’t focus on eating chocolate. |
| 1. Using sense of humour | Use of humour to manage emotions associated with the negative consequences of cancer and its treatment | 4 | 17 |  |
| *Finding humour in others' reactions §* | Not found in data |  |  |  |
| *Laughing about cancer and its consequences* |  | 3 | 13 | 003 Chris: For most people, most people diabetes is the worst thing you have to deal with and for me it’s like the least [laughs]. It’s so funny when I think about it like that it’s just ‘oh diabetes, fine’. [laughs]  018 Rosie: I’ve spoken about it with my friends. Not in a serious way. I try to laugh it off; do you know what I mean? But, when it all comes to play it’s a serious thing that might happen. |
| *Using humour to hide insecurities** |  | 2 | 8 | 003 Chris: Even today to this very day I like to make people laugh because it lets me hide my own insecurities under the laughter and I think that’s an important thing is just keep on and that’s what promotes the, but if I wasn’t able to do that it [my health] would have declined.  008 Hugh: I remember being at school and I’ve got all these scars and I always felt like people were looking at me and things like that and you think like I felt a little bit uncomfortable and you think… well I remember one time ‘oh yeah I was in Australia and I got mauled by a tiger and I won.’ I used to always lie. |
| 1. Using support | Use of appropriate supports to assist in one’s recovery and recuperation following treatment | **24** | **100** |  |
| *Companionship from pet* |  | 3 | 13 | 024 Daisy: Just having a dog, even that one act is just... he has made, I'll get emotional here, such a difference to my life. |
| *Drawing support from similar other* |  | 7 | 29 | 009 Imogen: The team I play for and everyone there is just lovely. I mean, they completely understand. Everyone’s in a different… We’re all in the same boat, we’re disabled, but I mean we’re all disabled for different reasons.  014 Nick: It’s just nice to have people that you know have gone through the same treatment and journey as you. |
| *Having someone to talk to** |  | 9 | 38 | 003 Chris: That’s all I want, it’s all I wanted for years, is someone I can just go and vent to and leave feeling that I’ve just drained out, out of many weeks, out of many months of shit.  011 Keith: I started talking about stuff slowly, and it’s kind of just helped me from there, you know, and started making me realise that I need to start talking about... if I started talking about the illness and started talking about the bullying, it releases a lot of pressure, which it did, so it kind of helped a little bit, you know?  012 Laila: I think it’s good to talk to people, personally I think like being be able to tell someone erm what you’ve been through. |
| *Receiving formal support* |  | 11 | 46 | 009 Imogen: I suffer with, like, severe anxiety when it comes to the hospital. I'm on two different tablets for it. I go see my GP every four weeks.  022 Beth: I did have a physiotherapist to get mobility in my arm again  024 Daisy: I mean psychiatrists, psychologists. I had a support worker as well who helped me with my anxiety a lot |
| *Receiving support from charities and organisations** |  | 5 | 21 | 009 Imogen: The Teenage Cancer Trust is absolutely brilliant. I mean they’ve got events every year, you know it's like seminars and conferences on general wellbeing after cancer. And they do surveys every year like, ‘What can we do to make your life better?’ They’re always there. |
| *Receiving support from educational provider** |  | 8 | 33 | 009 Imogen: I get such amazing support from even like the university.  017 Quinn: There was a guy who did classes with pupils who were a bit behind with their work or their behaviour wasn’t good. So, I went to a separate group for educational support. The guy that did that, as well, he would take me down to the gym on a Tuesday morning just to get some exercise and stay active, sort of thing. |
| *Receiving support from family*† |  | 20 | 83 | 003 Chris: They look after me, put me up, I don’t have to pay a fortune to live there. They feed me you know what I mean they pay for that. You know I have internet, I have telly. I have all these things that I probably couldn’t afford if I went out on my own.  012 Laila: My family, they know that I want to lose weight and like be more fit and that so they do help and even in the house they try and bring food that will not make me not want to do it they’ll bring healthy food and that snacks and that.  024 Daisy: I mean I was completely disassociating from what was going on around me. My parents would be like, ‘Come on, try and engage with us. Try and talk to us’. |
| *Receiving support from friends*† |  | 10 | 42 | 006 Freya: My friends have been really good and my family as well. I think maybe when you’re going through it things change a bit but now that like years have gone by its like nothing kinda happened really its quite good.  024 Daisy: They know when I can talk about things and then they know when not to ask me and when not to push me, without even me having to say anything. |
| *Receiving support from cancer care team** |  | 16 | 67 | 006 Freya: When I first finished we went a lot more to the consultant so if I had any issues or anything, it would be like every few months that I would see them so I could talk to them about that so it’s quite like nice to know that’s someone’s always there to check up on you if you have got anything you might be worried about.  011 Keith: I mean, I’ve always got [consultant] at the back of my head, talking to us. Yeah, basically talking to me in the back and saying, ‘You need to lose weight. You need to do this to keep yourself healthy and help yourself going’. |
| *Receiving support from partner*† |  | 5 | 21 | 010 Joanne: My partner is quite healthy himself so he’s kinda like someone to lean on every time I need a bit of help so if it wasn’t for the physical and normal relationship with a partner I think it would be a lot more harder to try and keep yourself motivated but my partner helps me. |
| *Receiving support in the workplace** |  | 2 | 8 | 008 Hugh: I had to do it and I had good people around that were showing me what to do and things like that even though I only lasted 2 months but me confidence took a big huge boost from that. |
| *Seeking formal help* |  | 12 | 50 | 001 Alice: Em I’d tried Slimming World, Weightwatchers em everything you could think of to try and lose weight and I just couldn’t… so I ended up going to my GP.  014 Nick: I’m finding things now much, much better. At first, I wasn’t coping well. I was seeing a counsellor. I was back and forth to my doctor, because I was very stressed out.  016 Pia: I got [the consultant], to do a blood test to see whether I could still have children and that. |
| *Seeking support from family*† |  | 7 | 29 | 024 Daisy: So like my parents I tell everything to and just tell them how I feel. |
| *Seeking support from friends*† |  | 7 | 29 | 008 Hugh: When I went to the gym before and that I got a lot more done because I felt like with someone it’s a bit of a competition and maybe I’ll get more out of it. Whereas to go by meself …you just think ‘I can’t do it’. I can’t like engage.  012 Laila: I always did feel left out so I like I started talking to one friend and she was like it’s good if you do tell everyone because they will like, not everyone just the main people, so they will understand.  022 Beth: I just try to reach out more, rather than waiting for someone to come to me, just realising that you’ve got to reach out. |
| *Seeking support from cancer care team** |  | 6 | 25 | 010 Joanne: Talking to the nurses at the hospital because they know what to say because they’ve probably been asked that question, like the same question at the exact same time like all over the day all over the week all over the year they’ve probably been asked the same question their whole career they have the best advice I’ve got to say. |
| *Seeking support from partner*† |  | 2 | 8 | 020 Tara: I try to speak to my partner as much as possible about it, if I worry I try and speak with him as much as I can. |

* new specific strategy identified in the CCSs data

† original specific strategy has been sub-divided into new categories

*§* original specific strategy not identified in CCSs data
